# Correspondence

**Published:** 1861-02

**Authors:** 


### Correspondence.

MESSRS. EDITORS:—The vulcanite controversy is becoming quite warm and exciting. The New York Dental Journal for January, is taking part in the discussion,—thus adding to the publicity and interest by new facts and disclosures—

makes it assume the shape of a triangular fight, and gives it the appearance of a "*discooshun wid sthicks.*"

I have read the controversy so far, including that lengthy rejoinder of Mr. Toland which he denominates, under certain circumstances, "a bore," and in view of the whole matter have been led to ask myself what many others no doubt have asked themselves or their friends, "What may all this be about? What is the motive on the part of the contestant, seeing that he is not a practitioner?" and in answering the question have been led to the conclusion that either he is fond of litigation for the sake of the excitement, or that he has a constitutional dislike to direct taxation where the necessity or right to impose such is not clear and indisputable.

Now, the first he would hardly be disposed to gratify at an expense of time and means such as it would necessarily entail, and therefore I incline to the latter as the true explanation or solution of the question. If I am correct in my conclusion as to motive, and the patentee can not substantiate his claim to the monopoly of this substance, then Mr. Toland will not only deserve, as he has already received, the thanks of the profession for his bold and determined resistance, but some more tangible evidence of their appreciation of his devotedness to, and sacrifices for their interests, and such he should receive promptly.

In the Philadelphia Directory for 1861, which is just out, the publishers have introduced a new feature, that of making a separate list of dental practitioners, under the head of "Dentists who have received the degree of Doctor of Dental Surgery. (D. D. S.)" (The list, however, is unfortunately imperfect, containing names of practitioners in the city who are not graduates, omitting others who are.)

This is making a distinction which may or may not result in good, but I can see no just ground for complaint; for it is altogether a proper recognition of those who have devoted their time and means to the thorough acquirement of their profession; and the public, who are thus informed of the existence of such an institution as a Dental College, have a right to know who its graduates are. And again, it is to be hoped that these graduates will be spurred up to greater proficiency and thus reflect honor upon their alma mater; by all of which, the reputation and standing of the profession will be enhanced and the people come to recognize the importance and the true mission of the dentist.

The most that can be said against it is, that it may possibly provoke some bitterness and antagonism to the school, on the part of some of those who are not graduates—but I can not see why—and which of course is not to be desired; but there need be no fear that old practitioners will take offense, as their reputations were made before the school was in existence. I therefore conclude that good must result from this very public recognition of the existence of a dental college, and of its graduates in our community, and as it has more than a local interest, I note the fact here.

What progress is the Harris testimonial fund making? I have seen no report of progress as yet. It is every way desirable that every practitioner at home, also those in Europe, should have this matter urged upon their attention, that they may have the opportunity of contributing if so disposed; therefore, this notice. Yours, O. U. C.

*Philadelphia, January* 19, 1861.

